# Hepatitis C seroprevalence among people living with HIV/AIDS and pregnant women in four provinces in Cambodia: an integrated bio-behavioral survey

**DOI:** 10.1186/s12879-022-07163-2

**Published:** 2022-02-22

**Authors:** Phearavin Pheng, Laurence Meyer, Olivier Ségéral, Phalla Chea, Siyan Yi, Sovannary Tuot, John M. Kaldor, Vonthanak Saphonn

**Affiliations:** 1 University of Health Sciences, 73 Monivong Boulevard, Phnom Penh, 12201 Cambodia; 2 Ecole Doctorale de Santé Publique, Service de Santé Publique du GH Hôpitaux, Universitaires de Paris Sud, Université Paris-Saclay, Inserm CESP U1018, Paris, France; 3 KHANA Center for Population Health Research, Phnom Penh, Cambodia; 4 Saw Swee Hock School of Public Health, National University of Singapore and National University Health System, Singapore, Singapore; 5 Center for Global Health Research, Touro University California, Vallejo, CA USA; 6 Kirby Institute, UNSW Sydney, Sydney, Australia; 7 Department of Community and Global Health, Graduate School of Medicine, The University of Tokyo, Tokyo, Japan; 8 Faculty of Social Sciences and Humanity, Royal University of Phnom Penh, Phnom Penh, Cambodia

**Keywords:** HCV infection, Prevalence, People living with HIV/AIDS, Pregnant women, Cambodia

## Abstract

**Background:**

Understanding the extent of viral hepatitis burden in specific subgroups, such as pregnant women and people living with HIV/AIDS (PLWHA), and their geographic distribution is essential for evidence-informed policy and mobilizing resources for targeted treatment and prevention efforts. However, in Cambodia, the epidemiology of hepatitis C remains uncertain. We estimated the hepatitis C virus (HCV) burden and transmission risk factors among PLWHA and pregnant women attending antenatal care (ANC) in Cambodia.

**Methods:**

Between March and April 2016, we conducted a cross-sectional survey in four diverse geographical areas: the capital city of Phnom Penh and three provinces. We collected information on demographic characteristics and risk behaviors and performed HCV antibody (Anti-HCV) testing among pregnant women attending public ANC clinics and among those receiving HIV care at the hospitals. We computed the prevalence of HCV among the two population subsets and performed logistic regression analyses to identify risk factors associated with HCV antibody positivity.

**Results:**

Of 935 participants enrolled, 510 (54.6%) were pregnant women and 425 (45.4%) were PLWHA. Anti-HCV prevalence was significantly higher in PLWHA than in pregnant women (29/425, 6.8% vs 5/510, 0.9%, *P* < 0.001). Of the geographic regions, Preah Sihanouk province (Southwest) had the highest anti-HCV prevalence among PLWHA (12.0%, *P* = *0.031*). There was no significant geographic difference in anti-HCV prevalence among pregnant women. In multivariable analyses (data subset to PLWHA), HCV infection was significantly associated with having a family member positive for HCV (OR = 7.6 [95% CI: 1.01–57.84], *P* = 0.048) and a history of intravenous medication injection in the last 5 years (OR = 7.1 [95% CI: 2.79–18.10], *P* < 0.001).

**Conclusions:**

HCV infection is relatively common among Cambodian PLWHA, likely related to intravenous medication injection and intra-familial viral transmission. Systematic HCV testing and care among PLWHA (and possibly their family members) might be necessary. Setting up a surveillance system for HCV might also be beneficial for some geographical regions and populations.

**Supplementary Information:**

The online version contains supplementary material available at 10.1186/s12879-022-07163-2.

## Background

Hepatitis C virus (HCV) infection is a major public health concern, with an estimated 130–150 million chronic hepatitis C virus infection worldwide [1] and 350,000 deaths each year due to hepatitis C-related causes, mostly cirrhosis and hepatocellular carcinoma (HCC) [2]. The prevalence of HCV varies substantially around the world. The estimated global prevalence of HCV is 2–3% [3] and ~ 8% of pregnant women have HCV infection [1], with the highest prevalence in low- and middle-income countries (LMICs) in Africa and Southeast Asia [4]. The World Health Organization (WHO) Global Health Sector Strategy for Viral Hepatitis calls for the elimination of viral hepatitis by 2030, aiming at a 90% reduction in incidence and a 65% reduction in mortality. To reach this target, 90% of chronic hepatitis cases need to be diagnosed, and 80% of eligible cases treated [1].

HCV treatment with direct-acting antivirals (DAA), is generally curative and success rates in LMICs are similar to those in high-income countries (HICs). Improved access to HCV testing and better epidemiological surveillance data are important steps in increasing HCV case detection and identifying those eligible for treatment. In particular, serological surveys can improve the understanding of the distribution of HCV infection in the general population and identify specific high-risk groups.

Globally, about 2.9 million people living with HIV/AIDS (PLWHA) are co-infected with hepatitis C virus [1]. The major cause of morbidity and mortality among people co-infected with HIV and viral hepatitis is potentially related to liver diseases [5]. It is recommended that this population should be prioritized for screening and providing with an appropriate and effective treatment for both HIV and hepatitis. In Cambodia, addressing HCV infection in PLWHA could possibly be done through expending access to treatment for hepatitis C for people with HIV who are co-infected with HCV in the existing National HIV program. However, the epidemiology of hepatitis C remains uncertain, and prevalence data are limited with a wide variation in reported estimations among this population ranging from 7.6% [6] to 78.5% [7] and the data restricted in selective areas. The most at-risk population seems to be patients who have household member with liver disease, aged over 40 years old, possibly because of historical exposures to unsafe medical injections or transfusions [6–8].

In Cambodia, the HCV prevalence was high in people who inject drugs (30.6%) [9] and low prevalence were found among young adult aged less than 45 years old (0.6%) [10]. However, no data on the HCV prevalence among pregnant women are available in Cambodia. Due to the lack of appropriate laboratories and high cost of HCV testing and treatment, screening for HCV has not been routinely provided in health care settings including ANC clinics to prevent vertical transmission.

Despite there were a few studies on HCV prevalence among PLWHA in the past, the data have been limited to some selected areas (the Northwestern Cambodia and to a hospital-based cohort in the Capital city). Assessing viral hepatitis burden in other regions across the Cambodia to have broader understanding of the extent of viral hepatitis at the national level is essential to inform policy development and resource mobilization to address this infection.

In order to fill a knowledge gap in Cambodia, we undertook a study to estimate HCV burden and transmission risk factors in four provinces and two specific populations, PLWHA and pregnant women attending antenatal care (ANC) in Cambodia. This study attempted to assess the HCV prevalence rates in different regions across the country to have representative HCV prevalence rates for the overall population in Cambodia.

## Methods

### Study design

This study was an integrated bio-behavioral (cross-sectional) survey undertaken to assess the prevalence of HCV among PLWHA receiving HIV care at the ART clinics and pregnant women attending public ANC clinics in Cambodia.

### Study settings

This study was carried out between March and April 2016 in antiretroviral therapy (ART) sites for PLWHA and in ANC clinics for pregnant women, located in four diverse geographical areas, including the capital city of Phnom Penh and three provinces in the West (Battambang), the Northwest (Siem Reap) and the Southwest regions (Preah Sihanouk) of Cambodia.

### Eligibility criteria

Individuals were eligible for the survey if they were at least 18 years old and either living with HIV/AIDS and had visited the ART clinics or pregnant women who visited the public ANC clinics during the study period.

### Sample size, sampling and recruitment

We used Open Epi software version 3 to calculate the sample size. Assuming the prevalence of HCV is 5% among pregnant women and 10% among PLWHA, to achieve a 2% margin of error for the pregnant women and 3% for PLWHA, power fixed at 80%, at 5% alpha-level, a design effect of 1, and a non-response rate of 10%, 502 participants were required for pregnant women and 423 for PLWHA.

A two-stage cluster sampling method was employed to select the eligible participants. Firstly, the probability-proportional-to-size-sampling method was used to select 51 clusters (34/153 clusters for pregnant women and 17/21 clusters for PLWHA) from the selected geographical regions. A cluster was a health center for pregnant women and an ART clinic for PLWHA. Secondly, a consecutive sampling strategy was used to invite 15 pregnant women and 25 PLWHA from each cluster. Each participant signed the consent form if they agreed to participate in the study. The participants underwent face-to-face interviews using a structured questionnaire, following by a blood sample collection for HCV testing. Prior to data collection, all interviewers attended a training course to ensure they understood and followed standard procedures. The participants were only identified by a unique anonymous code (no identifiable information were collected). The code was use to link each participant to their sociodemographic information, their HCV results and their appointment sleep when they were back to the clinics to get their HCV results. The questionnaire was checked for completeness, accuracy and legibility, and validated questionnaires were sent to the University of Health Sciences (UHS) for data entry.

### Behavioral questionnaire

The questionnaire was used to collect information on socio-demographic characteristics of participants such as age, sex, marital status, occupation and education. Multiple-choice questions were used to assess participants’ knowledge, attitudes and practices related to HCV infection. The knowledge questions explored four broad dimensions: basic information about HCV infection, mode of transmission, vaccination and treatment availability. Information on potential risk factors of HCV infection included history of intramuscular and intravenous injections, blood transfusion, and cases of HCV in the household. For each question, participants were offered three response options: “No”, “Yes” and “Don’t know”.

### Laboratory testing

A 5 ml blood sample was drawn from participants for HCV testing. Samples were kept at room temperature (18–25 °C) for a maximum of 6 h and then sent to the UHS laboratory in Phnom Penh. After centrifugation, plasma specimens were stored at − 80 °C for analysis. HCV antibody testing was done with a third-generation (G3) HCV ELISA assay (MONOLISA ANTI HCV PLUS version 3, Bio-Rad), according to the manufacturer’s instructions. Specimens showing a signal-to-cutoff (SCO) ≥ 1 were considered as positive for the anti-HCV antibody. Those with a SCO value < 1 were considered negative. All samples with a SCO ≥ 1 were further tested to confirm chronic HCV infection with a commercial HCV RNA viral load (VL) assay (OMUNIS, Clapiers, France). All runs were done on Bio-Rad CFX96 real-time PCR machines (Bio-Rad). HCV genotyping was performed in NS5B gene, using an in-house semi-nested RT-PCR and ANRS protocol.

### Statistical analyses

The prevalence of HCV infection among pregnant women and among PLWHA were presented as percentages and 95% confidence intervals (CIs), computed using exact methods (for binomial distribution). Categorical variables were presented as frequencies and percentages, while continuous variables were described with means and standard deviation (SD). Comparisons of categorical variables between HCV+ and HCV− participants across different socio-demographic and behavioral risk factors were performed using chi-squared or Fisher’s exact tests accordingly. Multivariable logistic regression analysis was performed to identify risk factors associated with HCV antibody positivity. All non-collinear variables with a *P* ≤ 0.2 in bivariate analyses or considered to be potential confounders based on previous studies were included in the models. Data were managed and analyzed using STATA 16 (StataCorp, USA), and *p*-values less than 0.05 were considered statistically significant.

## Results

### Characteristics of study participants

The characteristics of the 935 study participants (510 pregnant women and 425 PLWHA) are summarized in Table 1. The mean age of the participants was 42.9 years (SD, 9.3) for PLWHA and 27.6 years (SD, 5.3) for pregnant women. Approximately half of participants in both groups reported not having formal schooling or having Less than or primary school (46.6% in PLWHA and 45.3% in pregnant women), about 98.0% of pregnant women and only 64.0% of PLWHA were married. More than half of participants in both groups were farmers/self-employed and around 40% reported ever migrated to another country or to other places.

**Table 1:** Characteristics of study participants by population group (n = 935), Cambodia, 2016

Characteristics		PLWHA (N = 425)		Pregnant women (N = 510)	
		n	%	n	%
Mean age in years (± SD)		42.95 (± 9.37)		27.68 (± 5.33)	
Age group, years					
≤ 30		28	6.59	363	71.18
31–40		137	32.24	142	27.84
> 40		260	61.18	5	0.98
Sex					
Male		189	44.47	–	–
Female		236	55.53	510	100
Education					
No formal schooling/less than or primary school		198	46.59	231	45.29
Secondary school		100	23.53	148	29.02
High school/University or higher		127	29.88	131	25.69
Marital status					
Never married		37	8.71	10	1.96
Currently married		272	64.00	499	97.84
Separated/divorced/widow		116	27.29	1	0.20
Main occupation					
Employee (Government/Non-government)		111	26.12	110	21.57
Retired/unemployed/home duties		76	17.88	129	25.29
Farmer/self-employed/others		238	56.00	271	53.14
Residential mobility					
Always live here		240	56.47	328	64.31
Ever migrated (to another country/to other places)		184	43.29	181	35.49
Geographic area					
Battambang (West)		75	17.65	165	32.35
Siem Reap (Northwest)		75	17.65	135	26.47
Preah Sihanouk (Southwest)		25	5.88	30	5.88
Phnom Penh (Central)		250	58.82	180	35.29

### Knowledge/attitudes on hepatitis C prevention and care

Participants indicated high levels of willingness to get tested for HCV and to go for further investigations or treatment if HCV test positive. Correct answers to questions about whether blood should be screened for hepatitis C before transfusion, and barbers should use new blades or safe equipment for ear and nose piecing were reported by more than 90% of participants in both groups (Table 2).

**Table 2:** Knowledge/attitudes on hepatitis prevention and care

Variables		PLWHA (N = 425)		Pregnant women (N = 510)	
		n	%	n	%
Willing to get tested for hepatitis C		420	98.82	509	99.80
Willing to go for further investigations or treatment if HCV test positive		423	99.53	509	99.80
Asking for or use a new/sterilized syringe or needle		344	80.94	396	77.65
Getting the blood screened for hepatitis C before transfusion		389	91.53	394	77.25
Asking barber to use new blades or for safe equipment for ear and nose piecing		394	92.71	399	78.24

### Healthcare and behavioral factors potentially related to hepatitis C infection

Within the past 5 years, high proportions of participants had received intravenous medication (77.6% in PLWHA, 72.1% in pregnant women) or intramuscular injection (46.1% in PLWHA, 82.1% in pregnant women), or visited the dentist (47.7% in PLWHA, 40.5% in pregnant women (Table 3).

**Table 3:** Healthcare and behavioral factors potentially related to hepatitis C infection

Variables	PLWHA (N = 425)		Pregnant Women (N = 510)	
	n	%	n	%
Healthcare and behavioral factors^a^				
Ever been admitted to a hospital or clinic	115	27.06	167	32.75
Ever received intra-muscular medication injection	196	46.12	419	82.16
Ever received intravenous medication injection	330	77.65	368	72.16
Ever received blood transfusion	12	2.82	4	0.78
Ever undergone any plastic surgery procedure	12	2.82	7	1.37
Ear/nose piercing or tattoo	10	2.35	21	4.12
Ever visited dental clinic for dental care	203	47.76	207	40.59
Ever used any illicit drug injection	2	0.47	0	0.00
Having a family member positive for HCV	31	7.29	20	3.92
HCV testing				
Ever tested for viral hepatitis infection	181	42.59	169	33.14
Ever tested for hepatitis C	85	20.00	27	5.29

^a^Healthcare and behavioral factors reported over the past 5 years

### Prevalence of HCV infection

HCV antibody prevalence was as high as (29/425) 6.8% [95% CI: 4.6–9.6] in PLWHA and was low (5/510) 0.9% [95% CI: 0.3–2.7]) in pregnant women. Of the 34 anti-HCV-positive individuals, 24 (70.6%) had detectable HCV RNA, with substantially higher proportions of chronic infection in PLWHA compared to pregnant women (23/29) 79.3% [95% CI: 60.2–92.0] vs. (1/5) 20.0% [95% CI: 0.5–71.6], *P* = 0.007. A phylogenetic analysis of 24 samples with a detectable HCV RNA showed that (11/24) 45.8% were genotype 1b; (1/24) 4.1% were genotype 2a and (8/24) 33.3% were genotype 6 (6:4.1%, 6a:4.1%, 6e:12.5%, 6q:8.3%, 6r:4.1%) and 16.6% failed to amplify. In PLWHA, anti-HCV prevalence generally increased with age, from (1/28) 3.5% [95% CI: 0.0–18.3] in the youngest group (18–30 years) to (21/260) 8.0% [95% CI: 5.0–12.0] in the oldest group (> 40 years old), but the difference was not statistically significant (*P* = 0.51). In pregnant women, the anti-HCV prevalence was (4/363) 1.1% [95% CI: 0.3–2.7] in the18–30 years age group and (1/142) 0.7% [95% CI: 0.0–3.8] in the 31–40 years age group (Additional file 1: Table S1).

**Fig. 1:**
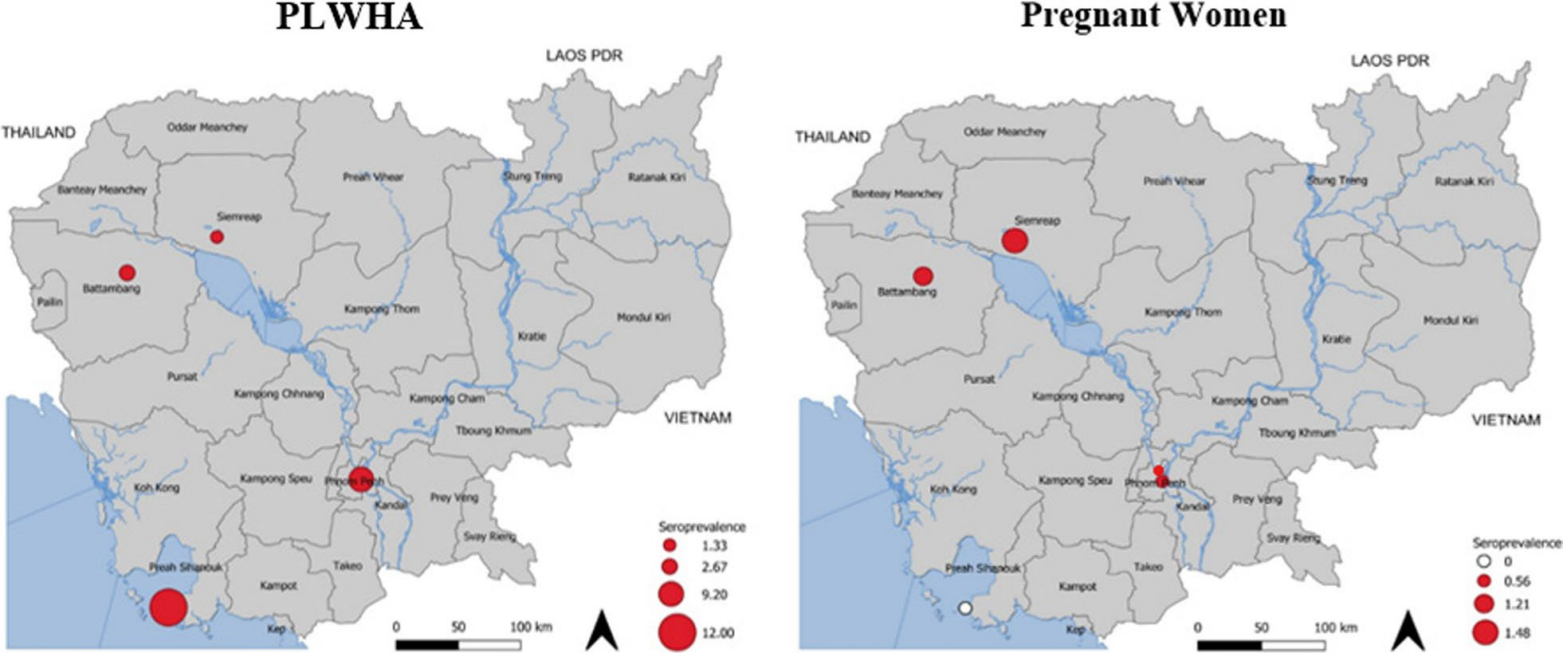
Geographical distribution of HCV prevalence among PLWHA (n = 425) AND pregnant women (n = 510) in Cambodia, 2016

We observed that in PLWHA group, Preah Sihanouk province (Southwest) had the highest anti-HCV prevalence among the four studied geographic areas: Preah Sihanouk province (3/21) 12.0% [95% CI: 2.5–31.2], Battambang (West) (2/75) 2.6% [95% CI: 0.3–0.9.3], Siem Reap (Northwest) (1/75) 1.3% [95% CI: 0.0–7.2], Phnom Penh (Central) (23/250) 9.2% [95% CI:5.9–13.4] (Fig. 1). There was no significant difference in anti-HCV prevalence across the four regions in pregnant women.

In PLWHA population, anti-HCV prevalence was significantly higher among those who had a history of intravenous medication injection in the past 5 years, at (28/330) 8.4% [95% CI: 5.7–12.0] compared to (1/95) 1.0% [95% CI: 0.0–5.7]) in those who had not. The same was observed in pregnant women, where all five HCV-infected women reported intravenous medication injection in the past 5 years (5/368) 1.3% [95% CI: 0.4–3.1] in those with this history vs. (0/142) 0.0% [95% CI: 0.0–0.2.5] in those without. In PLWHA, the anti-HCV prevalence was significantly higher in those who had a family member positive for HCV than in those who did not (9/31) 29.0% [95% CI: 14.2–48.0] vs. (20/394) 5.0% [95% CI: 3.1–7.7] (Table 4).

**Table 4:** Prevalence of Hepatitis C virus antibody per demographic characteristics of participants (n = 935), Cambodia, 2016

Characteristics	PLWHA (N = 425)		*P*	Pregnant women (N = 510)		*P*^a^
	Anti HCV positive			Anti HCV positive		
	n/N	%		n/N	%	
Education						
No formal schooling/less than or primary school	15/198	7.58	0.85	4/231	1.73	0.39
Secondary school	6/100	6.00		1/148	0.68	
High school/University or higher	8/127	6.30		0	0.00	
Marital status						
Never married	2/37	5.41	0.84	0	0.00	1.00
Currently married	20/272	7.35		5/499	1.00	
Separated/divorced/Widow	7/116	6.03		0	0.00	
Main occupation						
Employee (Government/Non-government)	8/111	7.21	0.59	0/110	0.00	0.71
Retired/unemployed/home duties	7/76	9.21		1/129	0.78	
Farmer/self-employed/others	14/238	5.88		4/271	1.48	
Ever received intra-muscular injection in the past 5 years						
No	12/229	5.24	0.16	0	0.00	0.59
Yes	17/196	8.67		5/419	1.19	
Ever received blood transfusion in the past 5 years						
No	28/413	6.78	0.83	5/506	0.99	1.00
Yes	1/12	8.33		0	0.00	
Ever visited dental clinic in the past 5 years						
No	12/222	5.41	0.23	4/303	1.32	0.65
Yes	17/203	8.37		1/207	0.48	
Ever received intravenous medication injection in the past 5 years						
No	1/95	1.05	0.01	0	0.00	0.33
Yes	28/330	8.48		5/368	1.36	
Having a family member positive for HCV						
No	20/394	5.08	< 0.001	5/490	1.02	1.00
Yes	9/31	29.03		0	0.00	
Geographic area						
Battambang (West)	2/75	2.67	0.031	2/165	1.21	0.810
Siem Reap (Northwest)	1/75	1.33		2/135	1.48	
Phreah Sihanouk ( Southwest)	3/25	12.00		0	0.00	
Phnom Penh (Central)	23/250	9.20		1/180	0.56	

^a^Fisher’s exact test

### Risk factors for HCV

Table 5 shows the results of crude and adjusted logistic regression on factors associated with HCV serological status among PLWHA. In bivariate analysis, participants who reported ever received intravenous medication injection in the last 5 years (OR = 8.7, 95% CI: 1.1–64.9, *P* = 0.04) and having a family member positive for HCV (OR = 7.6, 95% CI: 3.1–18.7, *P* < 0.001) were associated with an increased risk of HCV infection. In a multivariable model (adjusted for age and sex), HCV infection remained significantly associated with having a family member positive for HCV (OR = 7.6, 95% CI: 1.0–57.8, *P* = 0.048) and a history of intravenous medication injection in the last 5 years OR = 7.1 (95% CI: 2.7–18.1, *P* < 0.001). A multivariable analysis could not be performed among pregnant women due to the small number of HCV-infected women.

**Table 5:** Univariate and multivariate analysis of HCV risk factors for people living with HIV, Cambodia, 2016

Characteristics		Anti HCV positive		Unadjusted			Adjusted^a^		
		n = 29	%	OR (CI 95%)		*P*	OR (CI 95%)		*P*
Age group, years									
18–30		1	3.57	Ref.			Ref.		
31–40		7	5.11	1.45 [0.17–12.30]			1.42 [0.15–12.77]		
> 40		21	8.00	2.37 [0.30–18.33]		0.20	2.52 [0.30–20.88]		0.28
Sex									
Male		13	6.88	Ref.			Ref.		
Female		16	6.78	0.98 [0.46–2.10]		0.97	0.89 [0.38–2.03]		0.78
Ever received intravenous medication in the past 5 years									
No		1	1.05	Ref.			Ref.		
Yes		28	8.48	8.71 [1.17–64.91]		0.04	7.67 [1.01–57.84]		0.048
Having a family member positive for HCV									
No		20	5.08	Ref.			Ref.		
Yes		9	29.03	7.65 [3.12–18.75]		< 0.001	7.10 [2.79–18.10]		< 0.001

^a^Variables included in the multivariate model were: age, gender, history of intravenous medication injection, having a family member positive for HCV

## Discussion

To our knowledge, this study represents the first HCV seroprevalence survey conducted in four different provinces of Cambodia evaluating the burden of HCV infection and its risk factors in these two different groups of population (PLWHA and pregnant women).

For PLWHA, HCV seroprevalence was similar to that found in a hospital-based HIV cohort in Phnom Penh at the same calendar period (7.6%) [6]. The ani-HCV prevalence was slightly higher than that reported in Cambodia in 2014 [11] and in neighboring countries in a regional HIV cohort [12], excluding countries with a high proportion of people who reported that they injected drugs. Chronic HCV infection prevalence among HCV antibody positive was 79% among PLWHA our study, in line with the rates generally found in newly diagnosed HCV patients [13]. These results might be useful to guide the local strategy development in prioritizing the routine screening plan focusing on most at risk population for HCV case detection and identifying those eligible for treatment.

Our study showed that HCV seroprevalence among pregnant women was 0.9% [95% CI: 0.3–2.2]. This study could not provide the evidence of different distribution across the regions due to small number of positive cases. In a recent seroprevalence study conducted in Battambang province among the general population, anti-HCV prevalence was 0.6% [95% CI: 0.3–0.9] in adults 18–44 years old [14]. These results confirm the low anti-HCV prevalence (below 1%) in people under 40. The currently available literature shows that HCV seroprevalence in the general population reported in previous studies conducted in Cambodia varied over time with a decreasing trend in the most recent years, from 6.5% in 1993 [14] to 5.8% in 2012 [15] and 0.6% in 2018 [10]. The decrease in prevalence of HCV over time may be explained by a variety of reasons. Since 2000, there has been major improvements in clinical practice in the country, with emphasis on sterilization of medical equipment, use of disposable needles and systematic screening of donated blood, all of which contribute to the reduction of blood borne agents such as HCV [8]. It should also be noted that the availability of HCV testing and treatment, both in private sectors and public hospitals with the implementation of HCV program of Médecins Sans Frontières, could also have contributed to the decreasing prevalence of HCV by reducing the pool of infectious persons [10, 16]. In addition to that, the low anti-HCV prevalence among young age-group could be explained by the age cohort effect for which people in the old-age group were exposed to HCV longer than those in the young-age group. The decreased prevalence in younger population was also observed in Thailand [17, 18].

In multivariate analyses, ever received intravenous medication injection in the past 5 year and having a family member positive for HCV were strong, independent risk factors for HCV infection among Cambodian PLWHA, findings that are consistent with previous studies [6, 8, 10]. A history of intravenous therapeutic injection was a major risk factor since 33 out of the 34 anti HCV positive participants reported this practice in the past 5 years, as compared to 665/901 among the negatives. Therapeutic injections are a frequent practice in Cambodia as evidenced by our results showing that three-quarters of the participants, with both PLWHA and pregnant women reporting having received intravenous medication injection over the past 5 years. The overall treatment injection usage rate in Cambodia was 5.9 per person-year (women had a higher rate than men (7.5 vs. 4.3 per person-year), reported as one of the highest overall treatment injection usage rates worldwide [19]. Moreover, having an HCV-positive family member was an independent risk factor for HCV infection. HCV familial clustering and intra-familial viral transmission have been demonstrated previously in a study by Indolfi et al. [20] and in a Chinese study [21]. In Cambodia, sharing hygienic products, such as shaving razors, toothbrushes and nail clippers or ear/nose piercing or tattoo are common due to ignorance about sanitation and lack of resources. Our results argue in favor of the need of HCV screening for the whole family if one of member is positive and more particularly for children who are currently mostly unaware of access to HCV care programs. The risk factors of HCV infection could not be analyzed in pregnant women due to sample size issue. Of note, all five HCV-infected pregnant women also reported a history of intravenous medication injection in the previous 5 years.

HCV genotype 1b, 2a and 6 variants were identified. HCV genotype 1b (47.8%) and 6 (30.4%) were predominant in the current study. This finding is consistent with several previous studies in Cambodia, which reported HCV genotype 1b and 6 as the common genotypes observed in Cambodia [6, 15, 16, 22, 23]. The genotype 6 variant was also found as the most common in Vietnam and Laos [22], in blood donors in some regions of Thailand [16] and in intravenous drugs users in China [17, 24, 25].

### Limitations of the study

This study has several limitations. Our study attempted to assess the anti-HCV prevalence rates in different regions across the country to have representative anti-HCV prevalence rates for the overall population in Cambodia. However, our small sample size in Preah Sihanouk (South-western region) may limit the generalizability of the results for this region. Our findings may have been subject to recall bias or social desirability bias resulting in the underreporting of some risky behaviors and practices over the last 5 years such as the experience of injecting illicit drugs. Last, due to the small number of HCV positive in pregnant women, the risk factors of HCV infection in this group could not be assessed. However, in this study, we can rule out differential misclassification for assessing the risk factors of HCV infection linked to the history of risky behaviors and practices, since interviews were done before the result of HCV serology was known.

## Conclusions

In conclusion, we report a high HCV seroprevalence in PLWHA in Cambodia, likely related to past intravenous medication injection and/or intra-familial viral transmission. Our results led the national Cambodia program to offer systematic HCV testing in PLWHA and underline the necessity of care of HCV positive populations, measures which had already been initiated by National Program for adults. Children of HCV-infected parents living in the same household are likely the next target for testing and envisaging care access.

The low prevalence of HCV infection in the population under 40 years old seems to be confirmed but the anti-HCV prevalence varied across geographical area and specific populations, suggesting the need to set up a surveillance system to assess the anti-HCV prevalence across different population groups.

## Supplementary Information

**Additional file 1**: **Table S1**: seroprevalence and viremic prevalence per sex and per age category (n = 935), Cambodia, 2016.

## Data Availability

The datasets used and/or analyzed during the current study are available from the corresponding author on reasonable request.

## Abbreviations

PLWHA: People living with HIV/AIDS
HIV: Human immuno-deficiency virus
HCV: Hepatitis C virus
ANC: Antenatal care
Anti-HCV: HCV antibody
HCC: Hepatocellular carcinoma
LMICs: Low-and middle-income countries
WHO: World Health Organization
DAA: Direct-acting agents
HICs: High-income countries
ART: Antiretroviral therapy
UHS: University of Health Sciences
SCO: A signal-to-cutoff
VL: Viral load
