# Supplementary material for: Hepatitis C seroprevalence among people living with HIV/AIDS and pregnant women in four provinces in Cambodia: an integrated bio-behavioral survey
Source: BMC Infect Dis. 2022 Feb 22;22:177. doi: 10.1186/s12879-022-07163-2 (PMC8862396; doi:10.1186/s12879-022-07163-2)
Supplement: Supplementary file 1 — Additional file 1: Table S1: seroprevalence and viremic prevalence per sex and per age category (n = 935), Cambodia, 2016. [file 12879_2022_7163_MOESM1_ESM.docx]

Additional file 1

Table  S1: seroprevalence and viremic prevalence per sex and per age category (n=935), Cambodia, 2016
